# Visual diagnosis: Rectal foreign body: A primer for emergency physicians

**DOI:** 10.1186/1865-1380-4-73

**Published:** 2011-12-07

**Authors:** Bobby Desai

**Affiliations:** 1 Department of Emergency Medicine, University of Florida, PO Box 100186, Gainesville 32610, FL, USA

## Abstract

We present a case that is occasionally seen within emergency departments, namely a rectal foreign body. After presentation of the case, a discussion concerning this entity is given, with practical information on necessity of an accurate and thorough history and removal of the object for clinicians.

## Case

A 39-year-old male presented to the Emergency Department with vague complaints of abdominal pain and constipation. He stated that the abdominal pain was dull and crampy in nature and generalized in distribution. Furthermore, he stated that he had not had a bowel movement in 2 days, though he felt as if he had to have one. He denied constitutional complaints of fevers, chills, nausea, and vomiting, and denied urinary complaints as well.

**Figure 1:**
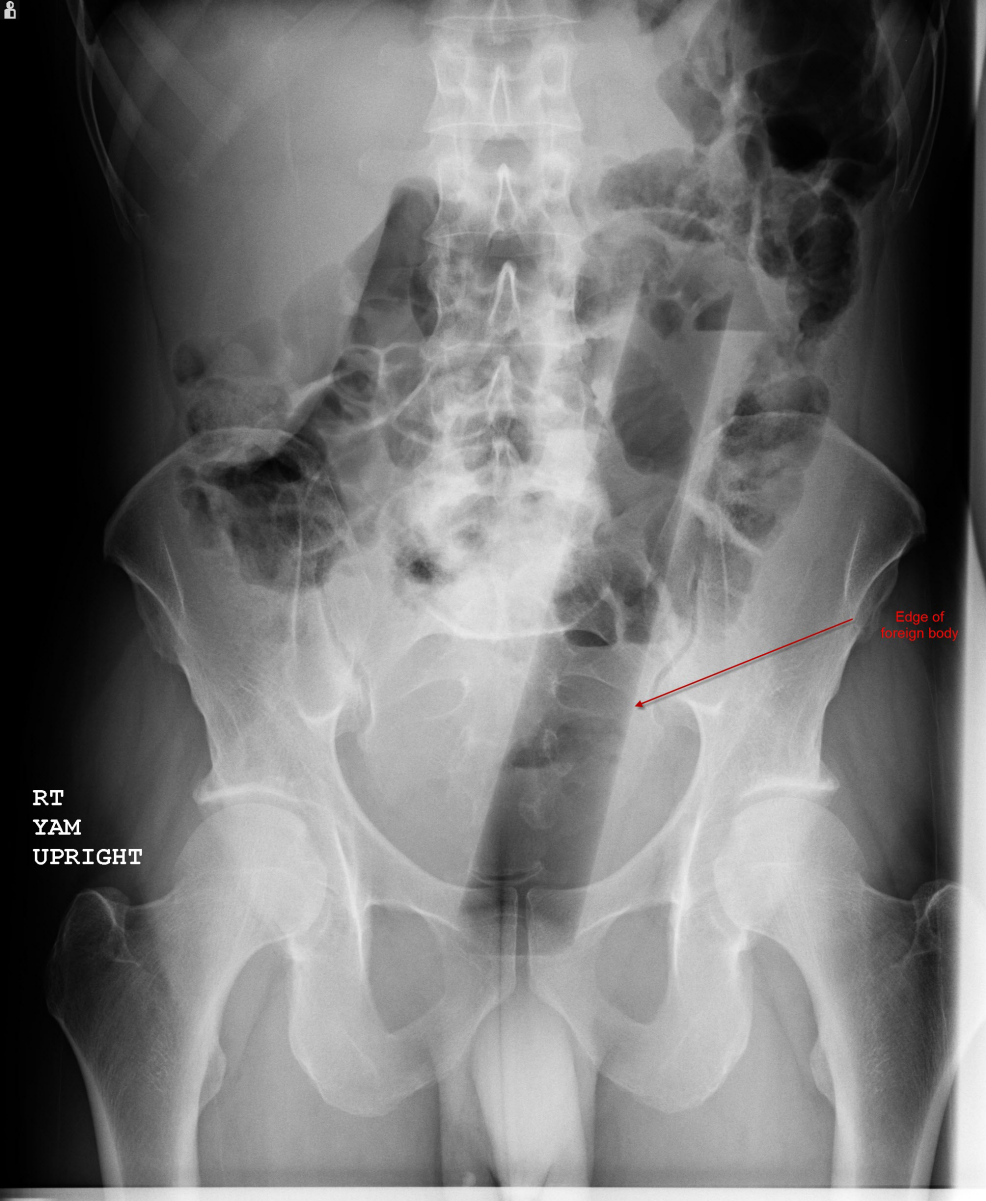
**AP view**.

**Figure 2:**
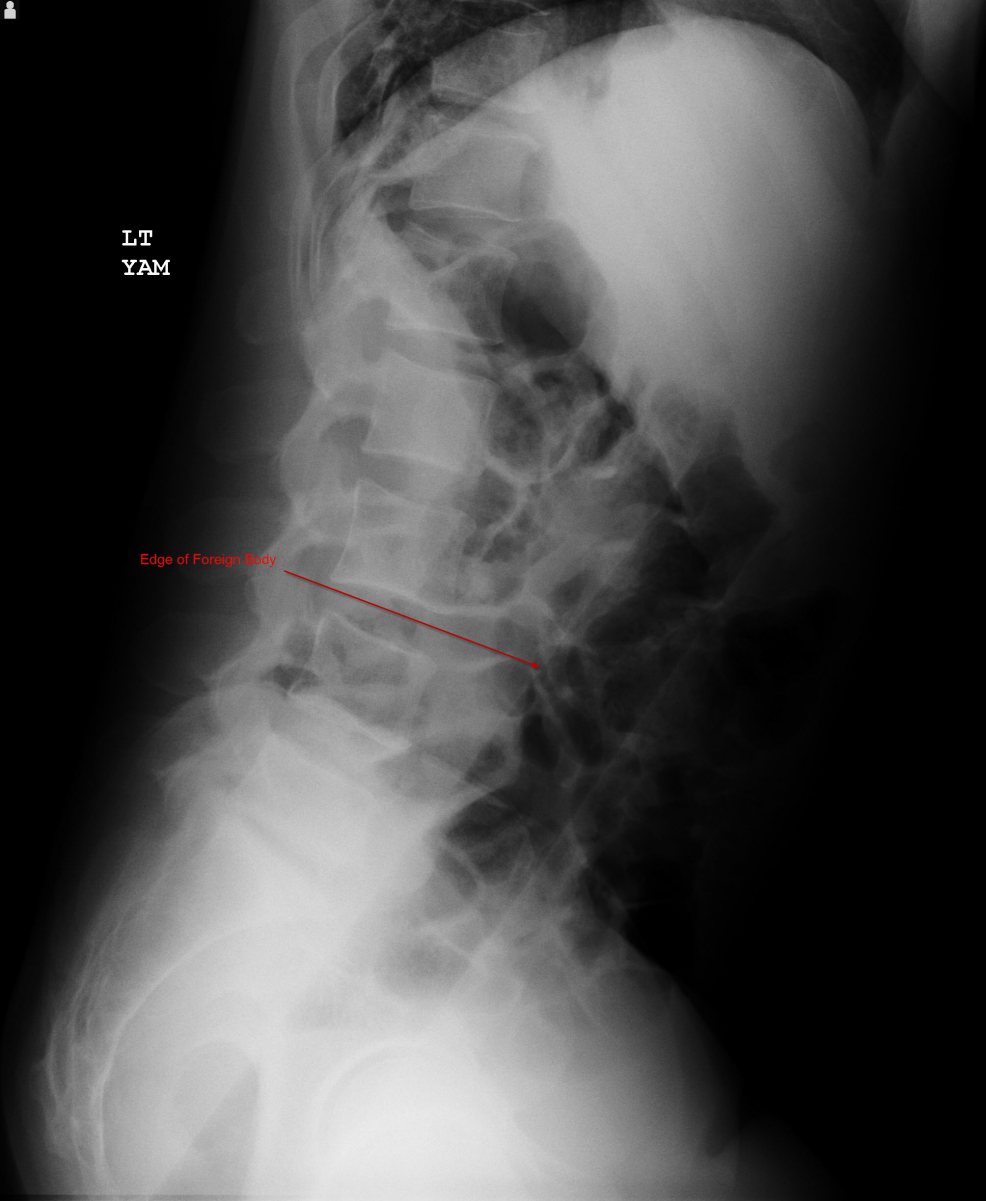
**Lateral view**.

The patient's vital signs were: temperature 37.2°C, pulse 87 beats per minute, respiratory rate of 20 per minute, and blood pressure 130/84 mmHg. The patient was awake, alert, and oriented to time, person, and place. His head, neck, cardiovascular, respiratory, and neurologic exams were all documented as within normal limits. His abdominal exam revealed a flat abdomen, diffusely tender with bowel sounds in all four quadrants. The physician noted a palpable mass in the left lower quadrant. Upon further examination, the mass felt "very hard" and had an "oblong" shape according to the physician notes. The patient was subsequently re-questioned about a family history of cancer, which the patient denied. The physician subsequently ordered basic laboratory tests and an abdominal X-ray. The AP and lateral X-rays are shown in Figures 1 and 2.

After obtaining the X-rays, the physician presented the X-rays to the patient and asked him what the object was. According to documentation, the patient replied that he did not know. The patient was subsequently placed in the left lateral decubitus position and an anoscope inserted. The object could not be visualized, and therefore no attempt was made to remove it. General surgery was consulted to see the patient and decided to take him to the operating room for removal. The patient agreed to this.

The object was noted to be the extension arm of a vacuum cleaner. It was removed according to notes with some difficulty and the patient was admitted to the hospital for observation and intravenous antibiotics. The patient was subsequently discharged 2 days later in excellent condition. Upon social work discharge, he was again asked how that apparatus managed to be placed where it was. The patient vehemently denied sexual assault or abuse, and insisted he did not know how it came to be there. He met no criteria for a mandatory psychiatric hold, but was offered the services of psychiatry, which he refused.

## Discussion

The majority of rectal foreign bodies seen in practice today are a result of deliberate insertion into the anal canal [1,2]. However, some sharp rectal foreign bodies that have traversed entire digestive tract may become impacted within the rectum, though this is far less common. These may typically present acutely with signs and symptoms of trauma, such as bleeding and perforation. In those instances where the object has had some delay either in presentation or diagnosis, the patient may present with signs and symptoms of infection - fever, chills, and sepsis. An abscess is likely to be found in these patients [3].

The majority of rectal foreign bodies have inserted purposefully by the patient themselves or by a sexual partner. These foreign bodies are usually blunt and take the shape of male genitalia [4,5]. Patients that repeatedly place these types of objects within the anal canal over time find that due to the increasing laxity of their rectal tone, they can insert objects of a higher caliber. These may be difficult for the patient to remove. Victims of sexual assault may present with objects of varying caliber, and these may not necessarily be of a blunt type. These patients require careful examination to ensure that perforation has not occurred. Drug mules have been known to either swallow latex balloons or directly place them within the anus.

Due to the sensitive nature of the complaint, it is occasionally difficult to elicit a history of the present illness. Furthermore, patients may be too embarrassed to present early to an Emergency Department. Common presenting complaints included abdominal pain, rectal pain, rectal bleeding, and constipation. For those patients who may have a bowel perforation, signs and symptoms of this may be present, including severe guarding, rebound tenderness, and fever, and these patients may present septic [6].

The physician should make every effort to ensure the patient feels comfortable during the history because of the necessity of gaining accurate information about the foreign body. Information should be sought as to the objects approximate size, shape, material, length of time since insertion, and any attempts at removal.

For examination, the patient should be placed in either the lateral decubitus position or lithotomy position. However, if the clinician suspects sharp foreign objects, a plain abdominal X-ray should be obtained first prior to examination to lessen the likelihood of inadvertent injury to either the patient or clinician. If sharp objects are noted, the exam should be deferred and surgery consulted. Furthermore, if there are signs and symptoms of bowel perforation, attempts at removal should cease and surgery should be consulted emergently as well. Plain abdominal X-rays are indicated in almost all cases; CT scans should be reserved for those with potential sepsis or equivocal peritoneal signs [3]. Hollow objects may have a gas pattern in their general shape. Radiolucent objects may require the use of rectal contrast; however, in these cases computed tomography may be the better modality to definitively diagnose the foreign body.

If this is not the case, the examination may proceed with a general survey of the anal area, noting fissures, excoriations, lacerations, and hemorrhoids. A digital rectal exam followed by anoscopy may reveal the object or signs of trauma proximal to the anal verge.

Treatment entirely depends on the location of the foreign body. Low-lying foreign bodies by definition are within the rectal ampulla, can often be palpated, and potentially can be removed in the emergency department [7]. High-lying objects usually require consultation as these are located proximal to the recto-sigmoid junction and require endoscopy for removal [7]. Due to the curvature of the sigmoid, these objects typically are unable to pass beyond this area [8].

Prior to attempting removal, the physician should consider medication with agents that relax not only the patient, but the anal sphincter as well. If the patient can tolerate the procedure without procedural sedation, they may be able to assist the physician by performing the Valsalva maneuver [9]. Regional anesthesia may be considered using a perianal block, though most emergency physicians will have limited experience with this [10].

Removal may be accomplished by having the patient perform the Valsalva maneuver while the physician applies pressure to the suprapubic area while simultaneously trying to grasp the foreign body through the anus. Either a finger or forceps may be used; forceps would be ideal if the object has a graspable edge. To improve visualization, an anoscope or other type of retractor may be used. If the object cannot be removed in this fashion, a Foley catheter may be used. A standard Foley usually cannot be used because of its inherent flexibility, and it often times may be difficult to pass the Foley past the object because of the object's diameter or length. Therefore, it is recommended that a three-way Foley catheter with a large balloon be used. A well-lubricated catheter is advanced past the object and the balloon inflated. If a three-way Foley is unavailable, a small-diameter endotracheal tube can be used. In either case, the catheter with the balloon inflated or the endotracheal tube is then slowly withdrawn. However, care must be taken not to force either tube past the object because of the risk of iatrogenic perforation. Two Foley catheters can be utilized if the object tapers near its distal end.

Complications of removal include hemorrhage, perforation, and mucosal tears [3]. Most experts agree that routine sigmoidoscopy should be undertaken for all patients subsequent to foreign body removal [6,7]. The emergency physician should observe the patient for signs of perforation after removal. The length of observation entirely depends on patient presentation and subsequent clinical status post-extraction.

## Consent

Written informed consent was obtained from the patient for publication of this case report and any accompanying images. A copy of the written consent is available for review from the Editor-in-Chief of this journal.

## Competing interests

The author declares that they have no competing interests.

## Authors' contributions

BD wrote, edited, and revised the entire report.
